# Interleukin-6-174G/C Polymorphism Contributes to Periodontitis Susceptibility: An Updated Meta-Analysis of 21 Case-Control Studies

**DOI:** 10.1155/2016/9612421

**Published:** 2016-12-06

**Authors:** Junfei Zhu, Bin Guo, Min Fu, Wushuang Guo, Yifang Yuan, He Yuan, Suhan Zhang, Haiyang Yu

**Affiliations:** ^1^State Key Laboratory of Oral Diseases, Sichuan University, Chengdu 610041, China; ^2^Institute of Stomatology of Chinese PLA General Hospital, 28 Fuxing Road, Haidian District, Beijing 100853, China; ^3^West China Medical School, Sichuan University, Chengdu 610041, China; ^4^Department of Prosthodontics, West China Hospital of Stomatology, Sichuan University, Chengdu 610041, China

## Abstract

*Introduction*. Chronic Periodontitis (CP) is suggested to be related to gene variations. Present study aims to quantitatively estimate the association between interleukin-6- (IL-6-) 174G/C polymorphism and CP susceptibility.* Materials and Methods*. Pubmed, Embase, and Web of Science were searched up to May 2016. The meta-analyses were performed using STATA 12.0.* Results*. 21 studies were yielded. Significant associations were found under heterozygote comparison and dominant model in studies fulfilling HWE (GC versus GG: OR = 0.690, 95% CI = 0.560–0.849, *P* = 0.000; CC + GC versus GG: OR = 0.690, 95% CI = 0.568–0.838, *P* < 0.001); significant associations were found under heterozygote comparison and dominant model in Caucasian studies fulfilling HWE (GC versus GG: OR = 0.752, 95% CI = 0.577–0.980, *P* = 0.035; CC + GC versus GG: OR = 0.737, 95% CI = 0.576–0.944, *P* = 0.016); significant associations were found under allele comparison, heterozygote comparison, and dominant model in Brazilian population (C versus G: OR = 0.648, 95% CI = 0.497–0.845, *P* = 0.001; GC versus GG: OR = 0.621, 95% CI = 0.441–0.876, *P* = 0.007; CC + GC versus GG: OR = 0.649, 95% CI = 0.470–0.896, *P* = 0.009).* Conclusion*. IL-6 174 polymorphism is associated with CP susceptibility. In Brazilian and Caucasian population, IL-6 174 GG genotype plays as a risk factor to CP.

## 1. Introduction

Chronic Periodontitis (CP) is a chronic pathological condition characterized by the inflammatory gingiva and the destruction of tooth-supporting structures [1]. The development of CP is facilitated by the interaction of multifactorial bacterial infection and host immune response [2]. The genetic factors, especially those involved in the evolvement of body immune system, have been playing instrumental roles in the pathogenesis of CP [3]. So far, experimental evidences have suggested particular associations between CP and human genetic polymorphisms [4–6]. In this light, CP can be treated as a genotype-related disease and the individual phenotype is determined by the effect of genetic and environmental factors corporately [7].

IL-6 is a pleiotropic cytokine not only exerting immunological effect but also functioning in haematopoiesis, bone metabolism, and tissue regeneration [8, 9]. The associations between IL-6 and CP have been extensively studied because of the role IL-6 played in periodontal host immune reaction and alveolar bone metabolism [10]. The location of human IL-6 gene is reported in the short arm of chromosome 7 (7p21), consisting of 5 exons and 4 introns [11]. There are several polymorphisms in the promoter region of IL-6 like -174 G/C, -572 G/C, -597 G/A, and so forth [12]. Among them, -174 G/C of IL-6, which is also designated as rs1800795, has been confirmed to influence the transitional activity of IL-6 [13].

Although observational studies have been performed to investigate the association between IL-6 -174 G/C polymorphism and CP, the findings were inconsistent [5, 10, 11, 14–19]. Previous meta-analysis demonstrated by Song et al. proposed a link between IL-6 -174 G/C polymorphism and periodontitis susceptibility in Brazilian population [20]. Nevertheless, this meta-analysis assessed CP and aggressive periodontitis (AP) together, and only 11 studies for CP were involved. In recent years, multiple studies were published regarding the association between IL-6 -174 G/C polymorphism and CP [5, 15, 17, 21–26]. Therefore, in present meta-analysis, 21 studies were included to further discover the role of IL-6 -174 G/C polymorphism in the pathogenesis of CP. The object of the present study is to evaluate current studies with more diversified and accurate approaches to assess the relationships between CP susceptibility and IL-6 gene variations.

## 2. Materials and Methods

Present meta-analysis was performed in accordance with the PRISMA-P (preferred reporting items of systematic reviews and meta-analyses protocols) statement which was recommended for the establishment of a systematic review and meta-analysis [27].

### 2.1. Eligibility Criteria

The eligibility criteria were as follows: (1) case-control studies; (2) the case groups consisted of patients diagnosed with CP, and the control groups consisted of periodontally healthy individuals; (3) IL-6 -174G/C polymorphism were detected and sufficient data of genotype distribution were provided for the calculation of odds ratio (ORs) and corresponding 95% confidence intervals (CIs); (4) studies with no repeated data. Studies which could not reach the eligibility criteria were excluded.

### 2.2. Search Strategy

A systemic manuscripts search was performed from the electronic databases including Pubmed, Embase, and Web of Science up to May 2016. In addition, the reference lists of the selected manuscripts and related reviews were also manually searched and screened for a comprehensive search result. The search strategies were presented as follows.

*Search Strategies*

*Pubpmed*

 ((((＇＇Periodontal Diseases＇＇[Mesh] OR ＇＇Periodontitis＇＇[Mesh]) OR ＇＇Periodontics＇＇[Mesh]) OR (((periodontal disease[Title/Abstract] OR periodontitis[Title/Abstract]) OR periodontal pocket[Title/Abstract]) OR periodontal tissue[Title/Abstract])) AND (((((＇＇Interleukin-6＇＇[Mesh] OR Interleukin-6[Title/Abstract]) OR Title/Abstract[All Fields]) OR IL-6[Title/Abstract]) OR IL 6[Title/Abstract]) OR Interleukin 6[Title/Abstract])) AND (((((＇＇Polymorphism, Genetic＇＇[Mesh] OR ＇＇Genetic Variation＇＇[Mesh]) OR Polymorphism[Title/Abstract]) OR Polymorphisms[Title/Abstract]) OR Genetic Variation[Title/Abstract]) OR rs1800795[Title/Abstract])

*Embase*

 ＇periodontal disease＇/exp OR ＇periodontitis＇/exp OR ＇periodontics＇/exp OR ＇periodontal disease＇:ab,ti OR ＇periodontitis＇:ab,ti OR ＇periodontal pocket＇:ab,ti OR ＇periodontal tissue＇:ab,ti AND (＇Interleukin 6＇/exp OR ＇Interleukin 6＇:ab,ti OR ＇IL 6＇:ab,ti OR ＇rs1800795＇:ab,ti) AND (＇DNA polymorphism＇/exp OR ＇genetic variability＇/exp OR ＇polymorphism＇:ab,ti OR ＇polymorphisms＇:ab,ti OR ＇Genetic Variation＇:ab,ti)

*Web of Science*

# 1
  TS = (periodontal disease) OR
  TS = (periodontitis) OR TS = (periodontal pocket) OR TS = (periodontal tissue)
# 2
  TS = (interleukin-6) OR TS = (IL-6) OR TS = (interleukin 6) OR TS = (IL 6)
# 3
  TS = (polymorphism) OR
  TS = (polymorphisms) OR TS = (genetic variation)
#1 AND #2 AND #3

### 2.3. Study Selection and Data Extraction

For the study selection, titles and abstracts of the search results were screened firstly and the full-text paper screen was performed next according to eligibility criteria. The results were screened by two authors (JF Zhu and M Fu) independently and a third author (WS Guo) was consulted if any discrepancies existed. The following characteristics were extracted from the eligible studies by two authors (JF Zhu and M Fu) independently, and discrepancies were resolved through discussion as follows: (1) name of the first author and year of publication; (2) country and ethnicity; (3) group size; (4) smoking status; (5) gender ratio comparability; (6) methods of genotyping; (7) type of the controls; (8) genotype distribution; (9) Hardy-Weinberg equilibrium (HWE) for the controls.

### 2.4. Quality Assessment

The methodological quality of the included studies was evaluated by two authors (JF Zhu and M Fu) independently. Newcastle-Ottawa scale (NOS) was applied for the quality assessment of the included case-control studies. The composition of NOS includes 3 chapters which are “Selection” (0–4 points), “Comparability” (0–2 points), and “Exposure” (0–3 points). For the chapter “Comparability,” ethnicity and gender were selected as the factors to be matched in present study. The final scores were calculated ranging from 0 to 9 [39]. Studies with scores of 0–3, 4–6, and 7–9 points were considered of low, moderate, and high quality, respectively [40].

### 2.5. Data Analyses

ORs and CIs were calculated to evaluate the association between IL-6 polymorphism and CP. Heterogeneity was estimated by *χ* ^2^ and *I* ^2^, *I* ^2^ > 50% and *P* < 0.05 was considered as significant heterogeneity then the random-effects model was used; otherwise, a fixed-effects model was used to assure the statistical efficiency [41]. The following four genetic models were applied for the meta-analyses of the IL-6 174 G/C polymorphism: (1) allele comparison (C versus G); (2) heterozygote comparison (GC versus GG); (3) the dominant model (CC + GC versus GG); (4) the recessive model (CC versus GC + GG). In addition, the *χ* ^2^ method was used to assess the HWE for the control groups. Subgroup analyses were conducted according to HWE fulfillment, ethnicity, smoking status, gender ratio, and source of the control. Moreover, the following characteristics were included as covariates in the meta-regression to explore the potential sources of heterogeneity: HWE fulfillment, smoking status, gender ratio, source of the controls, and genotyping method, a characteristic was considered as the source of heterogeneity if *P* < 0.05. Publication bias was measured by Begg's and Egger' linear regression test. A significant publication bias was considered if *P* < 0.05. All of the statistical analyses were processed using the software STATA 12.0.

## 3. Results

### 3.1. Study Selection

**Figure 1:**
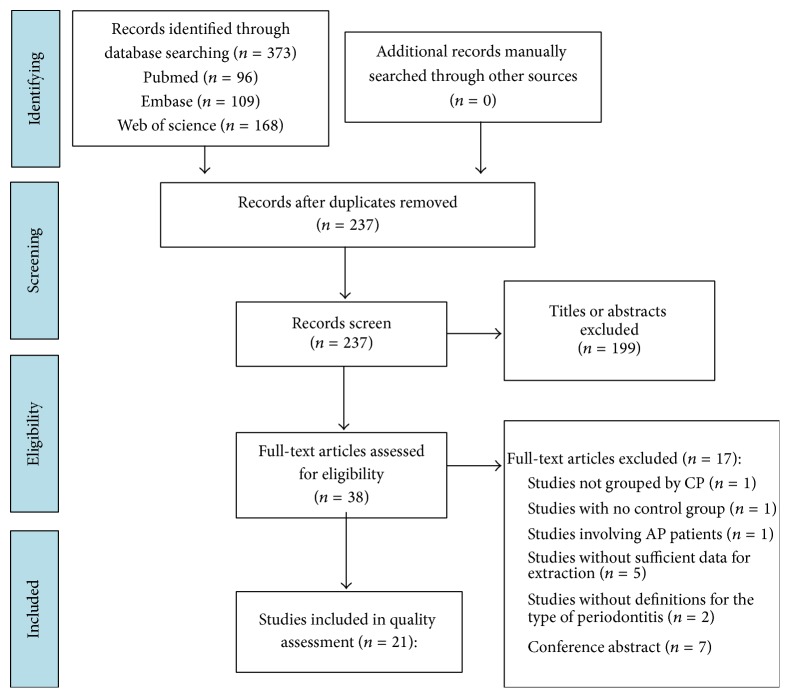
Selection process of the included study.

A total of 373 studies were yielded using the mentioned search strategies. After deleting the duplicates, record screen was performed. And 21 studies [5, 14–18, 21, 22, 24–26, 28–36, 38] involving 2422 CP patients and 3373 healthy individuals were identified to be eligible in present meta-analysis. The process of study selection and the reasons for exclusion are listed in Figure 1.

### 3.2. Characteristics and Quality Assessment

Of the 21 included studies, 10 studies were conducted in Caucasian population [5, 14, 17, 22, 28–30, 34–36], 6 studies were conducted in Brazilian population [15, 16, 21, 24, 26, 33], 4 studies were conducted in Asian population [18, 25, 32, 38], and 1 study was conducted in Indian population [31]. Seven studies involved nonsmokers as study subjects [5, 15, 22, 24–26, 31]. 12 studies involved case groups and control groups with matched gender ratio [14, 21, 24, 28–36]. Nine studies employed community controls [5, 14, 16, 18, 21, 24, 29, 32, 35, 38]. The control groups of 14 studies fulfilled the HWE [5, 14–16, 18, 21, 22, 24, 26, 29, 31, 33, 36, 38]. The scores of NOS ranged from 5 to 8. 12 studies were considered to be of high quality [5, 14, 16, 18, 21, 28, 29, 31, 32, 35, 36, 38] and 9 studies were considered to be of moderate quality [15, 17, 22, 24–26, 30, 33, 34]. The characteristics and NOS scores of the included studies were listed in Table 1.

### 3.3. Overall and Subgroup Analysis

**Figure 2:**
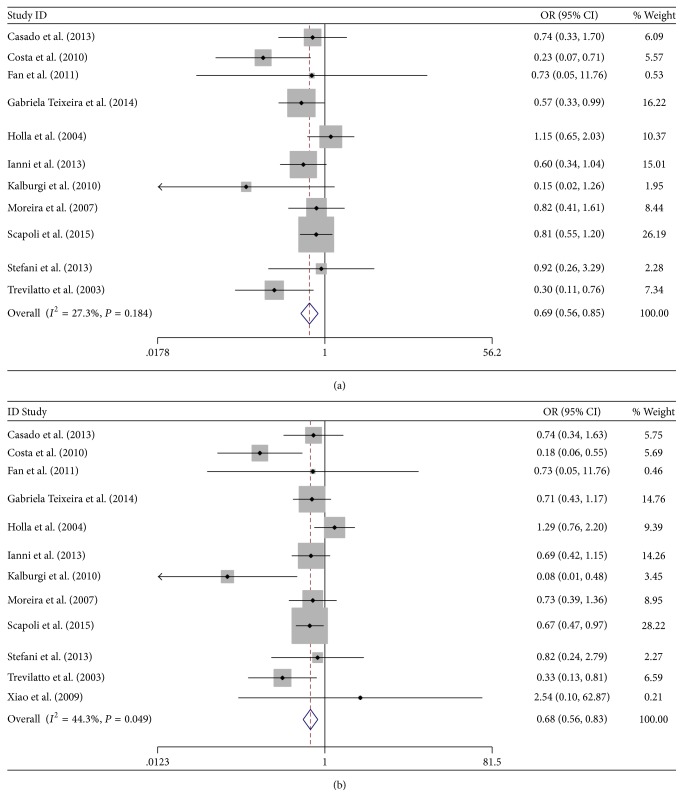
Heterozygote comparison and dominant model of studies fulfilling HWE. (a) GC versus GG (b) GC + CC versus GG.

**Figure 3:**
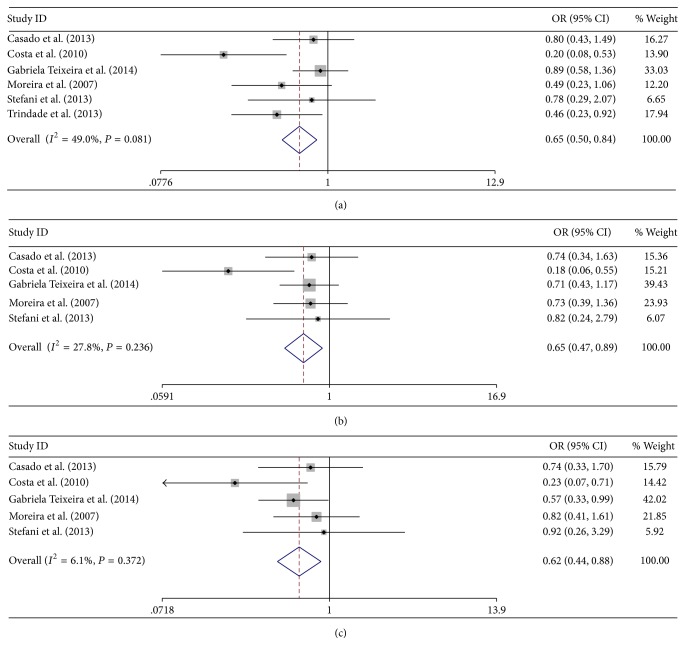
Allele comparison, heterozygote comparison, and dominant model of Brazilian subgroup. (a) C versus G; (b) GC + CC versus GG; (c) GC versus GG.

**Figure 4:**
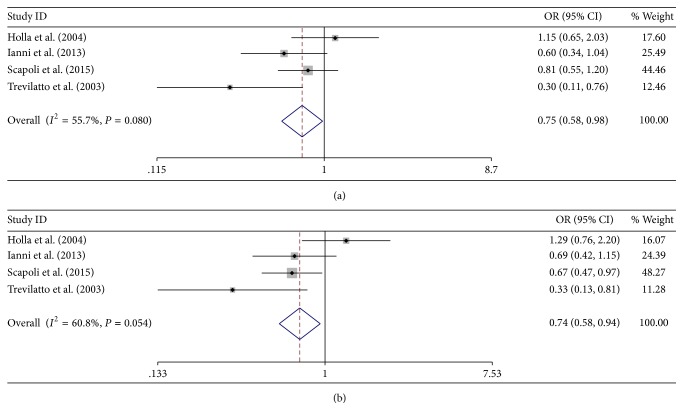
Heterozygote comparison and dominant model of Caucasian studies fulfilling HWE. (a) GC versus GG and (b) GC + CC versus GG.

For the overall analyses, no significant association was detected between IL-6 174 G/C polymorphism and CP under all of the 4 genetic models but significant heterogeneity was found under each genetic model. When stratified by HWE, significant results were found under heterozygote comparison and dominant model in studies fulfilling HWE (GC versus GG: OR = 0.690, 95% CI = 0.560–0.849, *P* < 0.001; *I* ^2^ = 27.3%  *P* = 0.184; CC + GC versus GG: OR = 0.690, 95% CI = 0.568–0.838, *P* < 0.001; *I* ^2^ = 44.3%  *P* = 0.049) (Figure 2). In the stratified analyses by race, no significant association was detected between IL-6 174 G/C polymorphism and CP in Caucasian population (C versus G: OR = 0.957, 95% CI = 0.670–1.366, *P* = 0.807; *I* ^2^ = 80.0%  *P* < 0.001; CC versus GC + GG: OR = 1.127, 95% CI = 0.495–2.565, *P* = 0.775; *I* ^2^ = 89.0%  *P* < 0.001; CC + GC versus GG: OR = 0.974, 95% CI = 0.640–1.482, *P* = 0.902; *I* ^2^ = 72.4%  *P* < 0.001; CC versus GG: OR = 0.806, 95% CI = 0.453–1.436, *P* = 0.464; *I* ^2^ = 67.2%  *P* = 0.003); significant associations were found under allele comparison, heterozygote comparison, and dominant model in Brazilian population (C versus G: OR = 0.648, 95% CI = 0.497–0.845, *P* = 0.001; *I* ^2^ = 49.0%  *P* = 0.081; GC versus GG: OR = 0.621, 95% CI = 0.441–0.876, *P* = 0.007; *I* ^2^ = 6.1%  *P* = 0.372; CC + GC versus GG: OR = 0.649, 95% CI = 0.470–0.896, *P* = 0.009; *I* ^2^ = 27.8%  *P* = 0.236) (Figure 3); significant association was found in Asian population under dominant model (CC + GC versus GG; OR = 2.692, 95% CI = 2.160–3.354, *P* = 0.000; *I* ^2^ = 0.0%  *P* = 0.830); significant associations were found under heterozygote comparison and dominant model in Caucasian studies fulfilling HWE (GC versus GG: OR = 0.752, 95% CI = 0.577–0.980, *P* = 0.035; *I* ^2^ = 55.7%  *P* = 0.080; CC + GC versus GG: OR = 0.737, 95% CI = 0.576–0.944, *P* = 0.016; *I* ^2^ = 60.8%  *P* = 0.054) (Figure 4). The results of overall and subgroup analyses were summarized in Table 2.

### 3.4. Metaregression

Metaregression showed that the HWE fulfillment was considered as the source of heterogeneity under heterozygote comparison and dominant model (GC versus GG: *P* = 0.004; CC + GC versus GG: *P* < 0.0001). The results of metaregression were summarized in Table 3.

### 3.5. Publication Bias

No significant publication bias was detected under allele comparison and recessive model. However, the Begg test and Egger linear regression test demonstrated the presence of publication bias with statistical significance under the heterozygote comparison and dominant model (GC versus GG: *P* = 0.035; CC + GC versus GG: *P* = 0.021).

## 4. Discussion

IL6 is known to exert an important effect in the pathogenesis of periodontitis [42]. During the development of CP, multiple biological actions could be mediated by the binding of IL-6 and its receptor (IL-6R), including hematopoiesis, angiogenesis induction, immunocyte activation, and osteoclast differentiation [43]. Evidence for the presence of IL-6 in serum, gingival crevicular fluid (GCF), and salivary suggested an altered production of IL-6 in patients with CP [44, 45]. A prolonged excessively-released IL-6 was reported to translate into persistence of oral inflammation and tissue destruction via proteases, osteoclasts, and methylation changes [46–48]. As a chronic inflammatory process caused by bacterial-induced host immune response, CP was proposed to involve genetic factors as determinants [3]. Holla et al. firstly suggested a relationship between IL-6 polymorphism and CP at position 572 [29]. Nibali et al. proposed a positive association between IL-6 174 GG genotype and the presence of* A. actinomycetemcomitans* and* Capnocytophaga sputigena* in subgingival plaque [49]. The studies performed by Raunio et al. and D'Aiuto et al. both demonstrated the links between IL-6 174 polymorphism and the presence of IL-6 in patients with severe periodontitis [4, 50]. In addition, the effect of IL-6 174 G/C polymorphism on the susceptibility of CP has been studied by multiple researchers, but the results of these disquisitions were inconclusive [5, 14, 15, 17, 18, 21, 28, 29].

To the extent of our knowledge, present meta-analysis of 21 studies quantitatively explored the association between IL-6 174 G/C polymorphism and CP. Our results provided evidence that IL-6 174 G/C polymorphism is associated with the susceptibility of CP.

As meta-regression suggested, the HWE fulfillment might be the effect modifiers for heterogeneity under heterozygote comparison and dominant model. In the overall analysis and Caucasian subgroup, stratified analyses for HWE lowered the heterogeneity and derived significant results under heterozygote comparison and dominant model, indicating IL-6 174 GG genotype might serve as a risk factor for CP. Beyond that, meta-regression analyses based on smoking status, gender ratio, source of control, and genotyping method have not explored any source of heterogeneity. Further studies based on the difference between population characterizes, such as family history, age, and other living habits, are encouraged to be developed [51].

As revealed by a large body of studies, the associations between genetic polymorphisms and certain diseases varied in different geographical regions and ethnic groups [52–56]. Herein, subgroup analyses based on the ethnicity were performed and the results of the Brazilian subgroup demonstrated insignificant heterogeneity under all of the 5 genetic models. The allele comparison of the Brazilian subgroup suggested that Brazilian CP patients are considered carrying less allele C compared with those periodontally healthy individuals. In addition, the analyses under heterozygote comparison and dominant model presented significant results, which suggested that the GG genotype also plays as a risk factor to CP in Brazilian population.

With regard to the Asian subgroup, present meta-analyses showed a significant result under the dominant model. However, for other genetic models, 2 of the 4 Chinese studies did not report genotype CC in their subject population [18, 38], so that only 2 studies were included in the homozygote comparison and the recessive model. In addition, significant heterogeneities were observed under other 3 genetic models. Pertinent disquisitions on the presence of IL-6 174 CC in Asian population remained conflicting. As suggested by a large body of studies regarding IL-6 174 G/C polymorphism, no genotype CC was found in Asian population [53, 57–61]. However, there are a number of studies indicating that IL-6 174 CC genotype can be detected in Asian individuals [62, 63]. Based on the inconsistency of the IL-6 174 CC distribution of the included Asian studies in present study, a definitive conclusion cannot be drawn in Asian population. Further studies are needed to elucidate the presence of IL-6 174 CC genotype in Asian population.

Shao et al. firstly performed the systematic review and meta-analysis based on the association between IL-6 174 G/C polymorphism and periodontitis in 2009 and demonstrated that GG genotype increased the risk of periodontitis, which was consistent with present findings [64]. In 2013, Song et al. updated this systematic review with lager sample size and stratified analyses based on ethnicity [20]. Compared with previous systematic review conducted by Song et al., present study included 10 more studies and focused on the association between IL-6 174 G/C polymorphism and CP; therefore the study conducted by Nibali et al. was excluded because it investigated CP and AP together [61]. The study performed by Franch-Chillida et al. was excluded for lacking of a classification for the type of periodontitis [65] In addition, NOS was employed for the quality assessment and meta-regression was applied to explore the source of heterogeneity. Moreover, although both present and previous studies reported the associations between IL-6 174 G/C polymorphism and CP in Brazilian population, the results were inconsistent. In the study conducted by Song et al. which included only 2 Brazilian studies, significant associations were found under the models “GG + GC versus CC” and “GG versus CC”, suggesting genotype CC lowers the risk to CP in Brazilian population. Yet present analysis tripled the amount of included Brazilian studies (*n* = 6) and demonstrated significant associations under model “C versus G,” “GC versus GG,” and “CC + GC versus GG,” which indicated that allele C play as a protection factor to CP, and genotype GG contributes to the susceptibility of CP. Those 2 findings were inequable but not paradoxical. With the accumulation of further evidences, we believe the association between IL-6 174 G/C polymorphism and CP susceptibility could be clearly elucidated.

The present study however has some limitations. First, although 21 studies were included, the quantity of the included studies was considered insufficient especially for the Asian and Indian subgroups. Additionally, several relevant studies could not be included in present meta-analysis owing to lacking of raw data [23, 66–69] or improper publication formats (Abstract) [6, 37, 70–74]. These might have reduced the number of the included studies and weakened the strength of the present results. Further, although subgroup analyses based on ethnicity were performed, the diversity in geography not only derived different ethnicity but also presented different living habits which exerted effect on CP. Therefore such confounding factors should be noticed when interpreting the results [13]. Moreover, evident publication bias was observed under the heterozygote comparison and dominant model, which might have distorted our present results because of the presence of the gray (unpublished) manuscripts.

In conclusion, within the limitations of this study, the present systematic review and meta-analysis supports that IL-6 174 polymorphism is associated with CP susceptibility. In Brazilian and Caucasian population, IL-6 174 GG genotype plays as a risk factor to CP. Further large-scale studies were required to validate our findings.

## Figures and Tables

**Table 1:** Characteristics of the included studies.

Author and Year	Country (ethnicity)	Type	Group size (case/control)	Smoke status	Control	Gender	Quality	HWE
Babel et al. 2006 [28]	Germany (Caucasian)	Case-control	124/116	Unknown	HC	Matched	7/9	Fulfilled
Brett et al. 2005 [14]	UK (Caucasian)	Case-control	106/99	Mixed	CC	Matched	7/9	0.000
Casado et al. 2013 [15]	Brazil (Brazilian)	Case-control	43/60	Unknown	HC	Not matched	6/9	0.805
Costa et al. 2010 [16]	Brazil (Brazilian)	Case-control	38/27	No smoke	CC	Not matched	7/9	1.000
Dosseva-Panova et al. 2015 [17]	Bulgaria (Caucasian)	Case-control	30/10	Unknown	HC	Not matched	5/9	0.035
Fan et al. 2011 [18]	China (Asian)	Case-control	178/130	Mixed	CC	Not matched	8/9	0.964
Gabriela Teixeira et al. 2014 [21]	Brazil (Brazilian)	Case-control	134/196	Mixed	CC	Matched	7/9	0.821
Holla et al. 2004 [29]	Czech (Caucasian)	Case-control	148/107	Mixed	CC	Matched	8/9	0.783
Ianni et al. 2013 [5]	Italy (Caucasian)	Case-control	77/278	No smoke	CC	Not matched	7/9	0.984
Jansson et al. 2006 [30]	Sweden (Caucasian)	Case-control	19/31	Unknown	HC	Matched	6/9	0.025
Kalburgi et al. 2010 [31]	India (Indian)	Case-control	15/15	No smoke	HC	Matched	7/9	0.217
Loo et al. 2012 [32]	China (Asian)	Case-control	440/850	Unknown	CC	Matched	8/9	0.000
Moreira et al. 2007 [33]	Brazil (Brazilian)	Case-control	155/54	Mixed	HC	Matched	6/9	0.106
Scapoli et al. 2012 [34]	Italy (Caucasian)	Case-control	177/117	Mixed	HC	Matched	5/9	0.000
Scapoli et al. 2015 [22]	Italy (Caucasian)	Case-control	284/211	No smoke	HC	Not matched	6/9	0.133
Stefani et al. 2013 [24]	Brazil (Brazilian)	Case-control	21/21	No smoke	HC	Matched	6/9	0.760
Tervonen et al. 2007 [35]	Finland (Caucasian)	Case-control	51/178	Mixed	CC	Matched	8/9	NA
Tian et al. 2013 [25]	China (Asian)	Case-control	122/532	No smoke	HC	Not matched	6/9	0.000
Trevilatto et al. 2003 [36, 37]	Brazil (Caucasian)	Case-control	48/36	Unknown	HC	Matched	7/9	0.142
Trindade et al. 2013 [26]	Brazil (Brazilian)	Case-control	49/60	No smoke	HC	Not matched	6/9	Fulfilled
Xiao et al. 2009 [38]	China (Asian)	Case-control	157/132	Mixed	CC	Not matched	7/9	0.502

HC = hospital control and CC = community control.

**Table 2:** Overall and subgroup meta-analyses.

Genetic models and subgroups	Number of studies	Association		Heterogeneity		Model of meta-analysis	Publication bias (*p*)
		OR (95% CI)	* P* value	*I* ^2^ (%)	* P* value		
C VS G	19	0.979 (0.714–1.344)	0.897	87.3	0.000	Random	0.517
HWE fulfilled	12	0.770 (0.558–1.064)	0.114	72.4	0.000	Random	
Caucasian	8	0.957 (0.670–1.366)	0.807	80.0	0.000	Random	
Brazilian	6	0.648 (0.497–0.845)	0.001	49.0	0.081	Fixed	
Asian	4	1.881 (0.667–5.305)	0.233	93.7	0.000	Random	
Indian	1	NA	NA	NA	NA	NA	
No smoke	7	1.032 (0.463–2.296)	0.939	94.7	0.000	Random	
Matched gender ratio	10	1.161 (0.869–1.552)	0.311	73.2	0.000	Random	
Community control	8	1.040 (0.760–1.424)	0.805	10.3	0.001	Random	
Caucasian (HWE fulfilled)	4	0.814 (0.559–1.184)	0.281	73.6	0.281	Random	

GC VS GG	18	0.897 (0.516–1.558)	0.699	89.8	0.000	Random	0.035
HWE fulfilled	11	0.690 (0.560–0.849)	0.000	27.3	0.184	Fixed	
Caucasian	8	1.066 (0.543–2.093)	0.852	84.9	0.000	Random	
Brazilian	5	0.621 (0.441–0.876)	0.007	6.1	0.372	Fixed	
Asian	4	1.673 (0.360–7.783)	0.512	89.1	0.000	Random	
Indian	1	NA	NA	NA	NA	NA	
No smoke	5	0.663 (0.506–0.871)	0.003	25.2	0.245	Fixed	
Matched gender ratio	10	1.211 (0.574–2.555)	0.615	91.4	0.000	Random	
Community control	8	1.120 (0.445–2.820)	0.809	93.0	0.000	Random	
Caucasian in HWE	4	0.752 (0.577–0.980)	0.035	55.7	0.080	Fixed	

CC + GC VS GG	19	0.907 (0.616–1.335)	0.621	83.9	0.000	Random	0.021
HWE fulfilled	12	0.690 (0.568–0.838)	0.000	44.3	0.049	Fixed	
Caucasian	9	0.974 (0.640–1.482)	0.902	72.4	0.000	Random	
Brazilian	5	0.649 (0.470–0.896)	0.009	27.8	0.236	Fixed	
Asian	4	2.692 (2.160–3.354)	0.000	0.0	0.830	Fixed	
Indian	1	NA	NA	NA	NA	NA	
No smoke	6	0.611 (0.274–1.361)	0.228	88.5	0.000	Random	
Matched gender ratio	11	1.034 (0.652–1.641)	0.886	81.0	0.000	Random	
Community control	9	1.801 (0.575–1.018)	0.952	85.2	0.000	Random	
Caucasian in HWE	4	0.737 (0.576–0.944)	0.016	60.8	0.054	Fixed	

CC VS GG + GC	17	0.846 (0.404–1.772)	0.657	92.5	0.000	Random	0.762
HWE fulfilled	10	0.896 (0.361–2.226)	0.813	86.5	0.000	Random	
Caucasian	9	1.127 (0.495–2.565)	0.775	89.0	0.000	Random	
Brazilian	5	0.834 (0.454–1.532)	0.559	40.0	0.155	Fixed	
Asian	2	0.996 (0.032–31.216)	0.998	99.2	0.000	Random	
Indian	1	NA	NA	NA	NA	NA	
No smoke	6	0.625 (0.157–2.488)	0.505	93.2	0.000	Random	
Matched gender ratio	12	0.888 (0.357–2.214)	0.799	91.8	0.000	Random	
Community control	6	1.009 (0.230–4.436)	0.990	95.0	0.000	Random	
Caucasian in HWE	4	1.788 (0.398–8.028)	0.448	94.2	0.000	Random	

NA = not available.

**Table 3:** Meta-regression analyses (*P* value).

Genetic model	HWE	Smoke	Gender	Method	Control
C versus G	0.121	0.832	0.224	0.805	0.539
GC VS GG	0.004	0.100	0.113	0.689	0.537
CC + GC VS GG	0.000	0.267	0.448	0.870	0.736
CC VS GG + GC	0.895	0.624	0.877	0.309	0.335
